# Peripheral Antinociception of a Chalcone, Flavokawin B and Possible Involvement of the Nitric Oxide/Cyclic Guanosine Monophosphate/Potassium Channels Pathway

**DOI:** 10.3390/molecules18044209

**Published:** 2013-04-10

**Authors:** Mohd Nasier Kamaldin, Muhammad Nadeem Akhtar, Azam Shah Mohamad, Nordin Lajis, Enoch Kumar Perimal, Ahmad Akira, Lee Ming-Tatt, Daud Ahmad Israf, Mohd Roslan Sulaiman

**Affiliations:** 1 Faculty of Medicine and Health Sciences, Universiti Putra Malaysia, Serdang 43400, Malaysia; E-Mails: mohd_nasier@yahoo.com (M.N.K.); azamshah@medic.upm.edu.my (A.S.M.); enoch@medic.upm.edu.my (E.K.P.); akira@medic.upm.edu.my (A.A.); mingtatt7286@yahoo.com (L.M.-T.); daud@medic.upm.edu.my (D.A.I.); 2 Faculty of Industrial Sciences & Technology, University Malaysia Pahang, Lebuhraya Tun Razak, Gambang 26300, Malaysia; E-Mail: nadeem409@yahoo.com; 3 Scientific Chairs Unit, Al-Jazeerah Building, Taibah University, Madinah al-Munawarah 41311, Saudi Arabia; E-Mail: nordinlajis@gmail.com

**Keywords:** flavokawin B, hyperalgesia, peripheral antinociceptive, nitric oxide, cyclic GMP, potassium channels

## Abstract

Previous studies have shown that systemic administration of 6'-hydroxy-2',4'-dimethoxychalcone (flavokawin B, FKB) exerts significant peripheral and central antinociceptive effects in laboratory animals. However, the mechanisms underlying these peripheral and central antinociceptive effects have yet to be elucidated. Therefore, the objective of the present study was to evaluate the participation of nitric oxide (NO)/cyclic guanosine monophosphate (cGMP)/potassium (K^+^) channels pathway in the peripheral antinociception induced by FKB. It was demonstrated that intraplantar (i.pl.) administration of FKB (150, 250, 375 and 500 µg/paw) resulted in dose-dependent peripheral antinociception against mechanical hyperalgesia in carrageenan-induced hyperalgesia test model in rats. The possibility of FKB having either a central or a systemic effect was excluded since administration of FKB into the right paw did not elicit antinociception in the contralateral paw. Furthermore, peripheral antinociception induced by FKB (500 µg/paw) was significantly reduced when l-arginine (25 µg/paw, i.pl.), Oxadiazolo[4,3-a]quinoxalin-1-one (ODQ; 50 µg/paw, i.pl.), glibenclamide (300 µg/paw, i.pl.), tetraethylammonium (300 µg/paw, i.pl.) and charybdotoxin (3 µg/paw, i.pl.) were injected before treatment. Taken together, our present data suggest that FKB elicits peripheral antinociception when assessed in the mechanical hyperalgesia induced by carrageenan. In addition, it was also demonstrated that this effect was mediated through interaction of the NO/cGMP/K^+^ channels signaling pathway.

## 1. Introduction

6'-Hydroxy-2',4'-dimethoxychalcone, also known as flavokawin B (FKB), is a naturally occurring chalcone present in many plants. It has been shown to exhibit various biological activities of important therapeutic potential, including anticancer [1,2], antinociceptive [3,4] and anti-inflammatory [5] properties. *In vitro* studies have also demonstrated that FKB inhibits nitric oxide (NO) and prostaglandin (PG) production in lipopolysaccharide (LPS)-stimulated murine monocytic macrophages (the RAW 264.7 cell line) [5,6]. The inhibition of both NO and PG production, which are products of the nitric oxide synthase (NOS) and cyclooxygenase (COX) pathways, respectively, has been reported as a potential therapy for different inflammatory diseases that involve major pro-inflammatory pathways. These diseases are highly targeted for anti-inflammatory and antinociceptive drug development.

In our continuous search for bioactive substances that possess antinociceptive activity, we previously demonstrated that systemic administration of a chalcone, FKB possessed significant antinociceptive effects against both chemical and thermal models of pain in mice and exhibited both peripheral and central analgesic activity [3]. In addition, we also suggested that systemic administration FKB exerts its antinociceptive effect via activation of the NO/cGMP/PKC/ATP-sensitive K^+^ channel pathway [4]. Despite the results of these studies, however, the exact mechanisms involved in the peripheral and central antinociceptive activity of FKB remains to be elucidated. A large body of research has demonstrated that the l-arginine/NO/cGMP/K^+^ channels pathway plays an essential role in the peripherally induced antinociception [7,8,9,10,11,12]. Thus, we hypothesized the peripheral antinociceptive activity of FKB could be due to the activation of this signaling pathway. Therefore, the present study was undertaken to investigate the peripheral antinociceptive activity of FKB in the carrageenan-induced hyperalgesia pain model in rats. Furthermore, efforts were also made to investigate the possible participation of the NO/cGMP/K^+^ signaling pathway in the peripheral antinociception induced by FKB.

## 2. Results and Discussion

**Figure 1:**
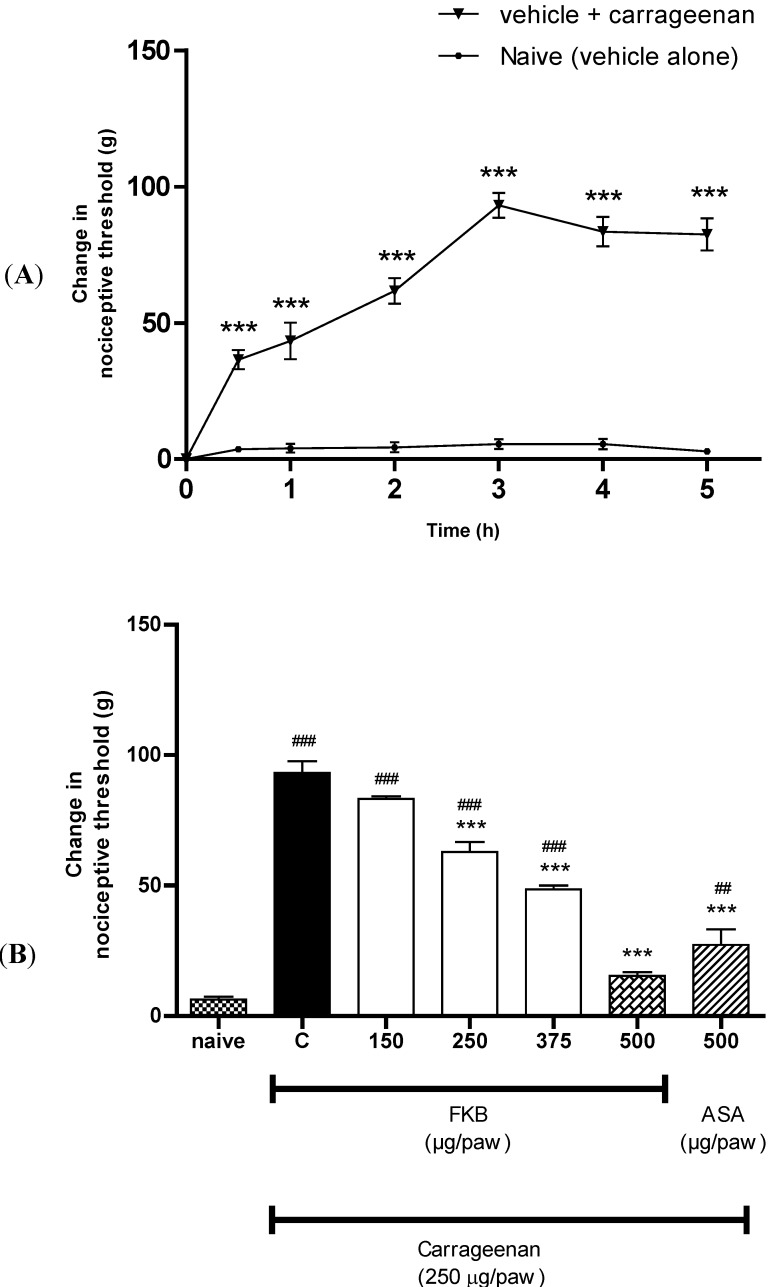
(**A**) Time course of hyperalgesia observed following local administration of carrageenan (250 µg/paw, i.pl.) in rats. Each point represents the mean ± S.E.M. (*n* = 6). *** *p* < 0.001 when compared to naive (vehicle alone) group (two-way ANOVA followed by Bonferroni *post hoc* test). (**B**) The effect of FKB (150, 250, 375 and 500 µg/paw, i.pl.) or ASA (500 µg/paw, i.pl.) on the nociceptive threshold in carrageenan-induced hyperalgesia in rats. FKB and ASA were administered 2 h 45 min following local administration of carrageenan (250 µg/paw, i.pl.). Each column represents the mean ± S.E.M. (*n* = 6). *** *p* < 0.001 when compared to control (C) group, ^###^ *p* < 0.001 when compared to naive group. (One-way ANOVA followed by Dunnett’s *post hoc* test).

**Figure 2:**
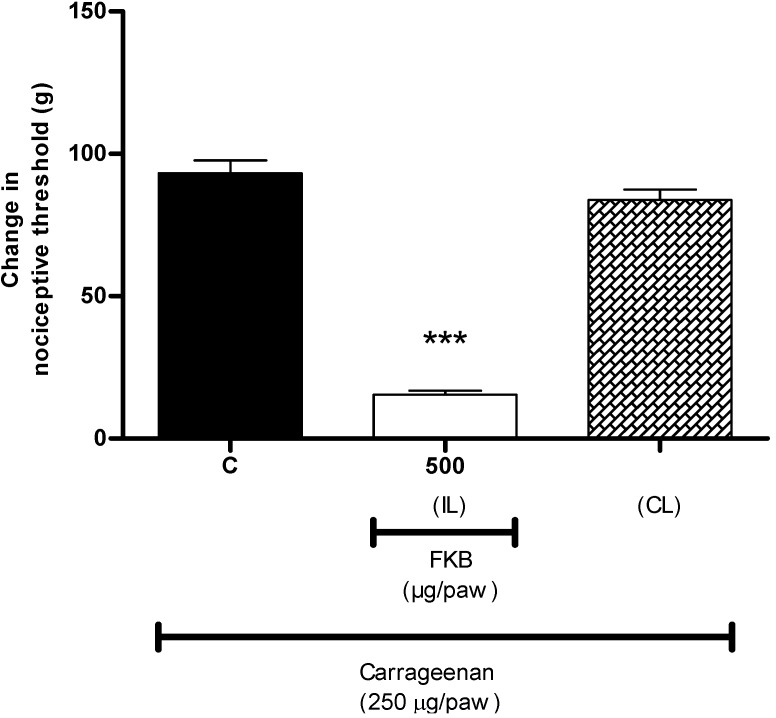
Exclusion of the central or systemic antinociceptive effect of FKB. FKB (500 µg/paw, i.pl.) was administered to the right hind paw 2 h and 45 min following carrageenan administration into both hind paws and nociceptive threshold was measured in the right (ipsilateral; IL) or left (contralateral; CL) hind paw. Each column represents the mean ± S.E.M. (*n* = 6). *** *p* < 0.001 when compared with control (C) group (One-way ANOVA followed by Dunnett’s *post hoc* test).

Previous studies have shown that systemic administration of FKB exerts dose-dependent antinociception when assessed using both chemical and thermal models of nociception in mice, suggesting the participation of both central and peripheral antinociception induced by FKB [3]. However, the mechanisms involved in the central and peripheral antinociception induced by FKB are yet to be elucidated. In the present study, using an acute model of inflammatory pain, we demonstrated the peripheral antinociceptive effect of FKB for the first time and its possible mechanisms of action. It was demonstrated that local administration of FKB induced dose-dependent inhibition of mechanical hyperalgesia induced by carrageenan. As shown in Figure 1A, i.pl. injection of carrageenan (250 µg/paw) into the right hind paw of rats evoked a significant mechanical hypersensitivity when compared with naive group (vehicle alone), with maximum effect observed at 3 h after carrageenan injection. Therefore, the time-point of 3 h after carrageenan injection was selected as a time of data recording of choice for all further experiments throughout the present study. The ipsilateral (right hind paws) i.pl. administration of FKB (150, 250, 375 and 500 µg/paw) significantly inhibited carrageenan-induced hyperalgesia in a dose-dependent manner, measured 3 h after i.pl. injection of carrageenan (Figure 1B) as compared with control group. ASA, used as a positive control drug, showed a similar significant inhibition of hyperalgesia. It was also demonstrated in the present study that FKB (500 µg/paw, i.pl.) did not inhibit the hyperalgesia evoked by i.pl. injection of carrageenan in the contralateral paw (left hind paw) (Figure 2), suggesting that the observed effect was restricted to the action of FKB at the local or peripheral level.

Carrageenan is commonly used as an inflammatory agent to study inflammatory hyperalgesia [13]. It is widely accepted that the local injection of carrageenan leads to inflammatory pain characterized by the presence of edema and hyperalgesia. These have been suggested to be initiated through the activation of peripheral nociceptors and the local release of various inflammatory mediators, such as histamine, serotonin and bradykinin, as well as prostanoids and cytokines; all of these are collectively involved in the sensitization of nociceptive pathways [13,14,15,16]. Based on the present results, it can be suggested that the peripheral antinociceptive effect of FKB in carrageenan-induced hyperalgesia could be due to the suppression of the production and/or release of these inflammatory mediators. Moreover, the results of the present study are in agreement with previous reports showing that FKB reduces nociception in several inflammatory pain models [3,4].

**Figure 3:**
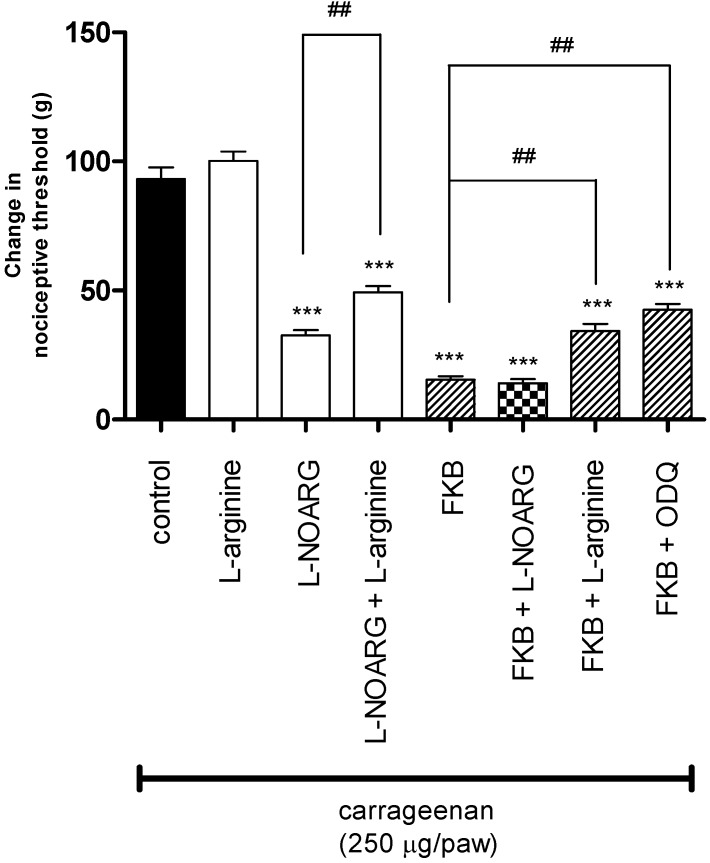
The effect of pre-treatment of animals with l-arginine (25 µg/paw, i.pl.), NG-nitro-l-arginine (l-NOARG; 35 μg/paw, i.pl.) or ODQ (50 μg/paw, i.pl.) on the peripheral antinociceptive effect of FKB (500 µg/paw) against carrageenan-induced hyperalgesia test in rats. Each column represents the mean ± S.E.M. (*n* = 6). *** *p* < 0.001 when compared with control group; *^##^* *p* < 0.01 when compared with l-NOARG-treated or FKB-treated group (One-way ANOVA followed by Dunnett’s *post hoc* test).

To look further into the possible peripheral antinociceptive mechanisms of FKB, the involvement of the l-arginine/NO/cGMP pathway was investigated in the carrageenan-induced hyperalgesia model in rats. The results in Figure 3 illustrate the participation of the l-arginine/NO/cyclic GMP pathway in FKB-induced peripheral antinociception using the carrageenan-induced hyperalgesia test. It shows that pre-treatment of rats with the NO precursor, l-arginine (25 µg/paw, i.pl.) significantly reversed the antinociception induced by peripheral administration of the NO synthase inhibitor, l-NOARG (35 μg/paw, i.pl.) or FKB (500 µg/paw, i.pl.). Likewise, the results depicted in Figure 3 show that the injection of a specific guanylate cyclase inhibitor, ODQ (50 μg/paw, i.pl.) also resulted in a significant reduction in the peripheral antinociception induced by FKB (500 µg/paw, i.pl.). These data support the hypothesis that FKB could produce its peripheral antinociceptive effect through the l-arginine/NO/cGMP signaling pathway, as is the case for several other analgesic drugs, such as morphine, diclofenac and dipyrone, which have been reported to induce peripheral antinociception through a similar mechanism [3,10,11,12,17,18,19,20].

Based on the data obtained in this study, we suggest that the local administration of FKB is, at least in part, linked to the inhibition of NO production and/or release, specifically via the inhibition of NO synthase activity. It is well-documented that an increase in the production of NO leads to an increase in the synthesis or release of various pro-inflammatory mediators that result in an increase of vascular permeability, the extravasation of fluid and protein and the development of nociception as well as hyperalgesia at affected sites [21,22]. In addition, the present results are also in agreement with our previous *in vitro* and *in vivo* studies demonstrating that FKB attenuates NO production [4,6]. NO is formed through the oxidation of the terminal guanidine nitrogen of l-arginine, which is then enzymatically converted into l-citrulline by NOS in mammalian cells.

The NO/cGMP pathway depends on the synthesis and release of NO triggered by the activation of NOS, which then activates the guanylate cyclase enzyme, guanylyl synthase, which is directly responsible for an increase in the intracellular level of the most important messenger of the system, cGMP [8,23]. It has been reported that cGMP plays an important role in the up- or down-regulation of nociceptors and is a key mediator of antinociceptive activity [4,7,23,24,25]. The involvement of cGMP in the peripheral antinociception of FKB in the present study was demonstrated as the specific guanylate cyclase inhibitor, ODQ, significantly attenuated the peripheral antinociception of FKB in the carrageenan-induced hyperalgesia model. ODQ blocks the ability of guanylate cyclase to form cGMP, but does not interfere with NO production [11]. Thus, in the present study, FKB may have inhibited GC and/or NOS, which was evoked by the local administration of carrageenan and thus provided a significant antinociceptive effect.

**Figure 4:**
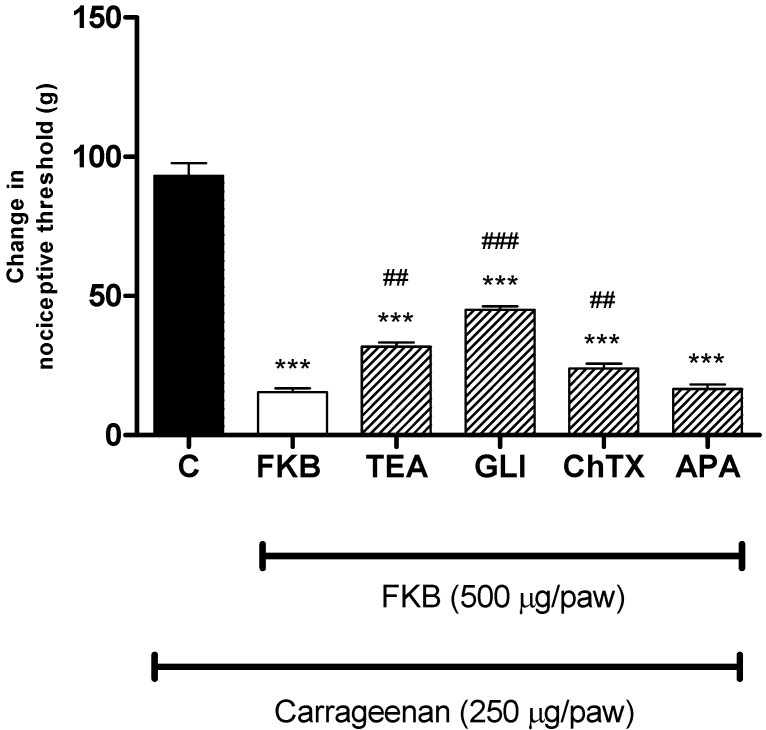
The effect of intraplantar administration of K^+^ channel blockers on the peripheral antinociceptive effect of FKB against carrageenan-induced hyperalgesia test in rats. Tetraethylammonium (TEA; 200 µg/paw), glibenclamide (GLI; 200 µg/paw), charybdotoxin (ChTX; 3 µg/paw) and apamin (APA; 3 µg/paw) were administered 15 min before FKB (500 µg/paw). Each column represents the mean ± S.E.M. (*n* = 6). *** *p* < 0.001 when compared with the control (C) group; *^##^* *p* < 0.01, *^###^* *p* < 0.001 when compared with the FKB group (one-way ANOVA followed by Dunnett’s post hoc).

There is now a substantial body of evidence indicating the relationship between the involvement of NO/cGMP pathway in the analgesia induced by certain drugs and the activation of K^+^ channels [17,19,26]. It is well known that K^+^ channels play essential roles in setting the resting membrane potential as well as controlling the excitability of neurons. The opening of K^+^ channels leads to hyperpolarization of cell membranes, that decrease cell excitability [4]. The results depicted in Figure 4 show that the pre-treatment of rat with a non-specific K^+^ channel blocker, tetraethylammonium (200 µg/paw, i.pl.), an ATP-sensitive K^+^ channel blocker, glibenclamide (300 µg/paw, i.pl.), and a large conductance Ca^2+^-activated K^+^ channel blocker, charybdotoxin (3 µg/paw, i.pl.) significantly reversed the antinociception caused by the peripheral administration of FKB (500 µg/paw, i.pl.). In contrast, the selective small conductance Ca^2+^-activated K^+^ channel blocker, apamin (3 µg/paw, i.pl.) failed to modify the peripheral antinociceptive effect of FKB in the carrageenan-induced hyperalgesia. Such results support that the antinociceptive effect caused by local administration of FKB in rats seems to involve the activation of voltage-gated, ATP-sensitive and large conductance Ca^2+^-activated K^+^ channels.

## 3. Experimental

### 3.1. Synthesis of the Compound

**Figure 5:**
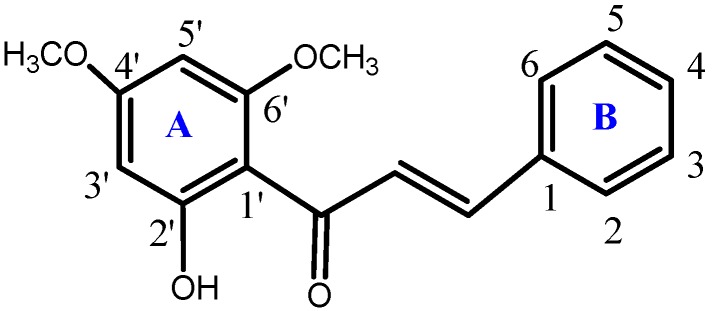
Chemical structure of flavokawin B (FKB).

6'-Hydroxy-2',4'-dimethoxychalcone or FKB (Figure 5) was synthesized and characterized at the Laboratory of Natural Products, Institute of Bioscience, Universiti Putra Malaysia as previously described [3]. The chemical purity of FKB was determined by GC/HPLC to be 99.9%.

### 3.2. Animals

These studies were conducted using male Sprague-Dawley rats (100–150 g), supplied by the animal house unit at the Faculty of Medicine and Health Sciences, Universiti Putra Malaysia. The animals were housed in groups of four per cage and maintained on a 12-h light/dark cycle. Standard laboratory food and tap water were made available *ad libitum* except during the experimental procedure. The animals were acclimatized and habituated to the laboratory environment for at least one week prior to the experiments and were used only once throughout the experiments. The experiments reported in this study adhered to the current guidelines for the care of laboratory animals and the ethical guidelines for experimental pain investigations in conscious animals [27], approved by the Animal Care Unit Committee, Faculty of Medicine and Health Sciences, Universiti Putra Malaysia. All efforts were made to minimize the number of animals and the intensity of noxious stimuli used to the level required to demonstrate consistent effects of the treatment. Each experimental group in the present study consisted of six animals.

### 3.3. Drugs

The drugs administered to rats were carrageenan λ, l-arginine hydrochloride, NG-nitro-l-arginine (l-NOARG), Oxadiazolo[4,3-a]quinoxalin-1-one (ODQ), glibenclamide, tetraethylammonium, charybdotoxin and apamin purchased from Sigma Chemical Co. (St. Louis, MO, USA). All drugs were dissolved in vehicle that consists of ethanol:Tween20:saline (5:5:90, v/v) and further subject to 5 min of sonication (Shanghai Kudos Ultrasonic Instrument Co. Ltd., China) to ensure complete dissolution of the chemicals. All drugs and FKB solutions were prepared immediately before the experiments were carried out and administered intraplantarly (i.pl.) at a volume of 50 µL per paw. The vehicle had no effects *per se* on nociceptive responses.

### 3.4. Measurement of Hyperalgesia

Peripheral anti-hyperalgesia activity was assessed using the Randall-Selitto paw pressure test, as previously described [11,28], with slight modifications. Briefly, hyperalgesia was induced by the injection of 50 µL of a carrageenan λ solution (250 µg/paw) subcutaneously into the plantar surface of the right hind paw of the rat. The mechanical nociceptive threshold was measured using a digital Randall Selitto paw pressure test apparatus (Model 2500, IITC Life Science, Woodland Hills, CA, USA). The device generates a mechanical force with a linear increase in force over time. The force was applied to the dorsal surface of the inflamed hind paw via a cone-shaped stylus with a rounded tip. The weight in grams (g) required to elicit nociceptive response, indicated by paw withdrawal, was defined as the nociceptive threshold. A cutoff force of 200 g was used to prevent damage to the paw. FKB or vehicle was given 2 h 45 min after carrageenan injection. The mechanical nociceptive threshold was again measured in the right hind paw and determined from the average of three consecutive trials recorded before (baseline) and at 3 h post carrageenan injection, which represented the maximum of effect of carrageenan. The hyperalgesia was calculated by the difference between these two means (change of the nociceptive threshold) and expressed in grams. The experimenter was blinded to the treatment of the animals.

### 3.5. Experimental Protocols

#### 3.5.1. Peripheral Anti-Hyperalgesic Effect of FKB

FKB (150, 250, 375 and 500 µg/paw), vehicle or ASA (500 µg) were administered intraplantarly in the right hind paw of the rat, 2 h 45 min following local injection of carrageenan into the ipsilateral paw. The nociceptive threshold was measured in the same paw before carrageenan injection (baseline) and 3 h after carrageenan injection. To determine whether FKB elicited its effect outside the injected paw (right), carrageenan was injected into both right and left hind paws, while FKB (500 µg/paw, i.pl.) was administered into the right hind paw 2 h 45 min after carrageenan injection. The nociceptive threshold was then measured in both the right (ipsilateral) or left (contralateral) hind paw.

#### 3.5.2. Participation of the l-Arginine/NO/cGMP Pathway

In order to determine the possible participation of the l-arginine/NO/cyclic GMP pathway in FKB-induced peripheral antinociception, right hind paws were pre-treated intraplantarly with a nitric oxide precursor, l-arginine (25 μg/paw, i.pl.), a non-selective NO synthase inhibitor, NG-nitro-l-arginine (l-NOARG; 35 μg/paw, i.pl.), or a guanylate cyclase inhibitor, ODQ (50 μg/paw, i.pl.), 1 h before the administration of FKB (500 μg/paw, i.pl.). In all experiments, FKB or vehicle was always administered 2 h 45 min after carrageenan injection and the nociceptive threshold was always measured in the right hind paw. Administration of these inhibitors by itself induced neither hyperalgesia nor antinociceptive effects (data not shown).

#### 3.5.3. Participation of the K^+^ Channels

In order to determine the involvement of K^+^ channels in the peripheral antinociception of FKB, rats were pre-treated with a non-specific K^+^ channel blocker, tetraethylammonium (200 µg/paw), ATP-sensitive K^+^ channel blocker, glibenclamide (200 µg/paw), large conductance Ca^2+^-activated K^+^ channel blocker, charybdotoxin (3 µg/paw) and small conductance Ca^2+^-activated K^+^ channel blocker, apamin (3 µg/paw) 15 min before they received FKB (500 μg/paw). In all experiments, FKB or vehicle was always administered 2 h 45 min after carrageenan injection and the nociceptive threshold was always measured in the right hind paw. Administration of these drugs alone neither induced any hyperalgesic nor antinociceptive effects (data not shown). The protocols used in the present study were obtained from the literature and based on our pilot experiments to determine the optimal dose and time point for the administration of each drug. 

### 3.6. Data Analysis

Statistical significance was determined by analysis of variance (ANOVA) with a *post hoc* Dunnett’s test (*p* < 0.05 was considered statistically significant). All data are presented as means ± SEM of measurements made on six animals in each group of separate experiments (*n* = 6).

## 4. Conclusions

In conclusion, taken together, the results obtained with the local administration of FKB strongly support the hypothesis that the peripheral antinociceptive activity of FKB is at least in part mediated through interaction of the NO/cGMP/K^+^ channels signaling pathway.
